# Editor's Letter-Box

**Published:** 1890-08-16

**Authors:** 


### Editor's Letter-Box.

[Our Correspondents are reminded that prolixity is a great bar to publication, and that brevity of style and conciseness of statement greatly facilitate early insertion.]

Sir,—I have read the article, "Tested by Results," in your issue of the 9th inst. with much interest, but am puzzled to understand what class of individuals you wish to indicate when speaking so bitterly of "money-makers." Kindly enlighten me.—I am, your obedient servant, J. H. C.

[We have no objection whatever to money-makers. What we object to is the selection of men for State honours on the sole ground of wealth, and the neglect of those who render really important services to the State, such, for example, as medical scientists and sanitarians.]

